# Oropouche fever cases diagnosed in Italy in two epidemiologically non-related travellers from Cuba, late May to early June 2024

**DOI:** 10.2807/1560-7917.ES.2024.29.26.2400362

**Published:** 2024-06-27

**Authors:** Concetta Castilletti, Antonio Mori, Andrea Matucci, Niccolò Ronzoni, Lukas Van Duffel, Giada Rossini, Pietro Sponga, Maria Luca D’Errico, Paola Rodari, Francesco Cristini, Ralph Huits, Federico Giovanni Gobbi

**Affiliations:** 1 Department of Infectious-Tropical Diseases and Microbiology, IRCCS Sacro Cuore Don Calabria Hospital, Negrar di Valpolicella, Verona, Italy; 2 Infectious Diseases Unit, AUSL Romagna, Forlì and Cesena Hospitals, Forlì, Italy; 3 Unità Operativa Complessa Microbiologia, IRCCS Azienda Ospedaliero, University of Bologna, Bologna, Italy; 4 Department of Medical and Surgical Sciences, Alma Mater Studiorum, University of Bologna, Bologna, Italy; 5 Department of Clinical and Experimental Sciences, University of Brescia, Brescia, Italy

**Keywords:** Oropouche fever, Oropouche virus, traveller, arbovirus, outbreak

## Abstract

Oropouche fever is caused by Oropouche virus (OROV), transmitted primarily through the bite of infected midges, particularly of the genus *Culicoides*. The virus is mainly circulating in Central and South America where several countries reported an ongoing outbreak. We report here two imported cases of OROV infection identified in Italy, late May–early June 2024. These cases indicate that in the shadow of a massive dengue outbreak in the Americas, the Oropouche outbreak might be more widespread than previously estimated.

Oropouche virus (OROV) was first detected in Trinidad and Tobago [1] but is currently spreading across Central and South America [2]. Outbreaks are frequent in the rainy season when the vector populations increase. The virus is transmitted to humans by biting midges and mosquitoes [2]. Here we describe Oropouche (ORO) fever cases in Italy in two travellers from Cuba, but not epidemiologically related.

## Case 1

A traveller in their mid-twenties without relevant prior medical history began experiencing diarrhoea and general malaise upon return to Italy on 26 May from a 2-week trip to Ciego de Ávila, Cuba. During their flight back, the patient developed high fever, accompanied by intense headache and nausea. On 27 May, the patient had severe arthralgia and marked retro-orbital pain. The patient consulted our hospital in Negrar, Verona. Upon examination, the patient was alert, oriented and cooperative. Lung auscultation revealed some fine crackles at the left base. A skin rash or exanthema was not observed. Physical examination was otherwise unremarkable.

The blood tests performed on the second day of fever onset showed leukopenia (white blood cell count 4,200/mm^3^ (norm: 4,300-10,800/mm^3^), with lymphocytes 800/mm^3^ (1,500-4,500/mm^3^), platelets 212,000/mm^3^ (150,000-400,000/mm^3^)), C-reactive protein (CRP) was 12 mg/L (norm:  ≤ 5 mg/L) and aspartate aminotransferase (AST) and alanine aminotransferase (ALT) were both 44 U/L (norm:  ≤ 35 U/L). The chest X-ray was normal.

Rapid tests for dengue for non-structural protein (NS) 1 antigen, dengue-specific immunoglobulin IgM and IgG antibodies (Standard F Dengue NS1 Ag FIA and SD Standard F Dengue IgM/IgG FIA, SD Biosensor, South Korea) were negative. Real-time reverse transcription PCR (RT-PCR) for dengue, Zika and chikungunya viruses (RealStar Chikungunya RT-PCR Kit 2.0, RealStar Dengue RT-PCR Kit 2.0 and RealStar Zika virus RT-PCR Kit 1.0, altona Diagnostics GmbH, Germany) from blood and urine samples were also negative.

## Case 2

An Italian traveller in their mid-fifties, with a history of hypertension, asthma and obesity, visited friends and relatives first in Havana and later in Santiago de Cuba from the beginning of May to the beginning of June 2024. The patient developed a fever 2 days after return to Italy, associated with retro-orbital headache and nausea, without myalgia or arthralgia. Three days later, during an emergency room visit of the hospital of Forlì (Emilia-Romagna region), arbovirus diagnostic was performed at the Regional Reference Center for Microbiological Emergencies (CRREM), Unit of Microbiology, IRCCS Azienda Ospedaliero-Universitaria di Bologna. Dengue NS 1 antigen (Standard F Dengue NS1 Ag FIA, SD Biosensor) and RT-PCR for dengue, Zika and chikungunya viruses (RealStar Chikungunya RT-PCR Kit 2.0, RealStar Dengue RT-PCR Kit 2.0 and RealStar Zika virus RT-PCR Kit 1.0, altona Diagnostics GmbH) on blood and urine samples were negative. Dengue IgM was negative whereas dengue IgG resulted weakly positive, IgM and IgG antibodies for chikungunya and Zika virus were also negative (Chikungunya VirClia IgG Monotest, Chikungunya VirClia IgM Monotest, Zika VirClia IgG Monotest and Zika VirClia IgM Monotest, VirCell Microbiologists, Spain).

As fever, headache and asthenia persisted, the patient was admitted on 11 June to the hospital of Forlì. The patient reported recent onset of febrile illness in close contacts in Santiago de Cuba. Physical examination was notable for slight paresis of the left arm with pronator drift, in the absence of neck stiffness or confusion. Lumbar puncture was not successful, and the patient declined further attempts. Computed tomography of the brain was normal, and the patient declined magnetic resonance imaging. Serial blood tests did not show any cytopenia or alterations of transaminases or kidney function. The highest CRP value measured was 8.8 mg/L (norm:  ≤ 5 mg/L). During further observation, diarrhoea developed and petechial lesions on the left leg were noted. Daily episodes of fever persisted until 14 June, after which all symptoms, except the left arm paresis, gradually abated.

## Molecular diagnostics for Oropouche virus

We performed an end-point RT-PCR specific for OROV targeting a partial fragment (approximate amplicon size 300 bp) of segment S of OROV on whole blood, serum and urine from both patients using OROV specific primers as previously described [3]. Whole blood and serum samples from Case 1 were positive, urine sample was negative; while for Case 2, whole blood and urine tested positive, serum sample was negative. We determined the sequence of the amplicons by the Sanger method. Both Case 1 and Case 2 sequencing products, submitted to GenBank (Basic Local Alignment Search Tool (BLAST) http://www.ncbi.nlm.nih.gov/blast/Blast.cgi; accession number: PP874910), clustered with OROV sequences in GenBank. Case 1 amplicon sequence showed 100% identity with OR500488.1 and 94.03% identity with the reference sequence (NC_005777.1).

## Discussion

Here we report about two epidemiologically non-related imported cases of ORO fever from Cuba to Italy. On 9 May 2024, Pan American Health Organization (PAHO) reported a surge in ORO fever cases outside the Amazon region, alongside reports of widespread dengue circulation [4]. Until week 18 2024, 5,193 confirmed cases of ORO fever had been notified in four countries in the Region of the Americas. On 27 May, the Ministry of Public Health of Cuba confirmed the presence of cases of ORO fever, in the province of Santiago de Cuba [5].

Oropouche outbreaks have been reported in the past in Brazil, Colombia, Ecuador, French Guiana, Panama and Peru [2]. The OROV is maintained in nature by two distinct transmission cycles. The virus has been detected in pale-throated sloths (*Bradypus tridactylus*), non-human primates and some wild birds involved in the sylvatic cycle. Humans appear to be the only vertebrate hosts in the urban transmission cycles, where the virus is transmitted by biting midges *Culicoides paraensis* (*Ceratopogonidae*) and by *Culex quinquefasciatus* mosquitoes (*Culicidae*) [2].

Oropouche virus is a virus of the genus *Orthobunyavirus* in the *Peribunyaviridae* family [6], belongs to the Simbu serogroup, which is composed by two phylogenetic subclades. Subclade A, which includes OROV and Manzanilla orthobunyaviruses, and subclade B, which includes Simbu, Shuni, Shamonda, Sathuperi, and Akabane viruses [7]. In the Americas, three OROV reassortants have been identified: Iquitos virus, Madre de Dios virus, and Perdões virus [6].

Oropouche fever has an incubation period of 3–10 days, and the illness is characterised by self-limiting fever, headache, muscle and joint pains and sometimes by photophobia, nausea or vomiting [2,6]. Occasionally, aseptic meningitis or meningoencephalitis have been described to follow OROV infections, but no fatalities have been reported. Oropouche fever typically has a biphasic course: with recurrence of fever after an acute phase of 2–4 days, the symptoms reappear 7–10 days later. Targeted antiviral treatment or vaccines against OROV infection are not available.

The Italian response to arboviruses is regulated by a 5-year National Plan for Prevention, Surveillance and Response to Arboviruses (PNA 2020–2025) [8]. Since 1990, Italy has an active surveillance system throughout the year for vector-borne diseases coordinated by Istituto Superiore di Sanità (ISS). In particular, the PNA applies to the One Health surveillance of arboviruses, with special reference to West Nile, Usutu, chikungunya, dengue, Zika, tick-borne encephalitis and Toscana viruses, as well as other arboviruses not subject to specific surveillance and response measures. Our hospital is designated by the Veneto Regional Directorate of Prevention, Food Safety, Veterinary, Public Health to perform active surveillance for arboviral infections in humans in support of the regional reference laboratory. Upon laboratory detection, both cases were immediately notified to the local authorities. On 5 June, a ProMED post was issued to alert clinicians, this post was published on 8 June [9].

The cases reported here represent to the best of our knowledge, the first documented importations of OROV to mainland Europe from the 2024 outbreak in some of the Central and South American regions. They illustrate the potential for the introduction of OROV in Europe via travel. *Culicoides* species are among the most abundant vectors of arboviruses in Europe and have presented challenges for veterinary public health, spreading Schmallenberg virus and bluetongue virus among livestock [10,11]. However, the vector competence of *Culicoides* species in Europe for OROV is unknown. Gaps in our understanding of the epidemiology of OROV complicate our assessment of the potential for development of transmissible viraemia in vertebrate hosts or the likelihood of autochthonous transmission in Europe. Our cases indicate that in the shadow of massive dengue outbreak in the Americas, the 2024 ORO fever outbreak may possibly be more widespread than previously estimated.

## Conclusions

We would like to emphasise the importance of expanding the travel history for considering a diagnosis of ORO fever in returning travellers from Central or South America, to include Cuba and other Caribbean countries. We recommend testing for OROV in travellers returning from these regions, who present with dengue-like symptoms but remain negative following adequate testing for dengue or other arboviral infections. Travellers to these regions should be advised to adhere to mosquito bite prevention measures, that include using of repellents containing N,N-Diethyl-meta-toluamide (DEET), IR3535 or icaridin, sleeping under repellent-treated mosquito nets and wearing protective clothing.
